# Development of the ERATbi App, a Clinical Decision Support System for Early Recovery After Traumatic Brain Injury in the ICU: Usability Study

**DOI:** 10.2196/79981

**Published:** 2026-02-06

**Authors:** Hsiao-Ching Yen, I-Hui Wu, Wei-Ling Hsiao, Sheng-Ru Lai, Chen-Hao Yang, Hsien-Chi Liao, Yin-Yi Han

**Affiliations:** 1 Division of Physical Therapy, Department of Physical Medicine and Rehabilitation, National Taiwan University Hospital, Taipei, Taiwan; 2 Department of Traumatology, National Taiwan University Hospital, No. 7, Chung Shan South Road, Taipei, 100, Taiwan, +886 2 2312 3456; 3 Department of Cardiovascular Surgery, National Taiwan University Hospital, Taipei, Taiwan; 4 Graduate Institute of Clinical Medicine, College of Medicine, National Taiwan University, Taipei, Taiwan; 5 Department of Nursing, National Taiwan University Hospital, Taipei, Taiwan; 6 Department of Dietetics, National Taiwan University Hospital, Taipei, Taiwan; 7 Division of Respiratory Therapy, Department of Integrated Diagnostics & Therapeutics, National Taiwan University Hospital, Taipei, Taiwan; 8 Department of Anesthesiology, National Taiwan University Hospital, Taipei, Taiwan

**Keywords:** brain injuries, traumatic, decision support systems, human engineering, intensive care units, rehabilitation, early, usability testing

## Abstract

**Background:**

Early rehabilitation in neurocritical care is often underutilized due to fragmented workflows, interdisciplinary coordination challenges, and the absence of structured digital decision support. Traditional clinical decision support systems (CDSS) often address single domains and lack adaptability to the dynamic, multiprofessional workflows of intensive care units (ICUs).

**Objective:**

To develop and evaluate the usability of the ERATbi App (Early Recovery After Traumatic Brain Injury App), a modular, tablet-based CDSS was designed to streamline early rehabilitation planning and strengthen interdisciplinary coordination for patients with moderate-to-severe traumatic brain injury (TBI) in intensive care settings.

**Methods:**

The ERATbi app integrates four functional modules—delirium risk management, precision nutrition, stepwise early mobilization, and respiratory care for rib fractures—into a unified interface. A simulation-based usability study was conducted with 18 ICU clinicians. Evaluation metrics included System Usability Scale (SUS) scores, task completion rates, error rates, and task durations. Additional user feedback was collected via a 5-point Likert satisfaction survey and semi-structured qualitative interviews.

**Results:**

The app demonstrated high usability (mean SUS score 83.6, SD 7.4), a 100% (18/18 participants) task completion rate, and a low error rate (4.2%). Average module completion time was 6.5 minutes, and user satisfaction was high (mean 4.7, SD 0.5). Users highlighted the value of the app’s visual logic, real-time alerts, adaptive thresholds, and modular workflow integration for enhancing team coordination and decision consistency.

**Conclusions:**

The ERATbi app demonstrated excellent usability, high user satisfaction, and clinical relevance in simulated ICU workflows. Its logic-driven, workflow- integrated design may support scalable, interdisciplinary implementation of early rehabilitation in neurocritical care settings.

## Introduction

### Epidemiology and Clinical Burden of Traumatic Brain Injury

Traumatic brain injury (TBI) is a major global public health concern and a leading cause of death and long-term disability worldwide [1]. In Taiwan, the annual incidence of hospitalized head injuries is approximately 126.1 per 100,000 population, equivalent to nearly 29,000 cases each year. Among these, an estimated 20.5% involve moderate-to-severe TBI, which is frequently associated with impaired consciousness, mechanical ventilation, and polytrauma [2].

### Barriers to Early Rehabilitation in the Acute Phase

Although the benefits of early rehabilitation are increasingly supported by evidence [3,4], its implementation in the acute phase remains limited. Major barriers include safety concerns, fragmented workflows, and the absence of structured clinical decision-support systems [5-8]. Clinical hesitation often stems from hemodynamic instability, fluctuations in intracranial pressure (ICP), and the complexity of managing multiple invasive devices. These challenges force clinicians to balance therapeutic benefits against physiological risks, contributing to variability in decision-making and delayed initiation of rehabilitation.

### Gaps in Multidisciplinary Integration and Decision Support

Early mobilization, nutritional therapy, delirium prevention, and respiratory management show positive impacts on outcomes of critically ill patients [9-15]. However, these interventions are typically delivered by separate disciplines with limited cross-team coordination, resulting in inconsistent implementation and delayed functional recovery. Most existing electronic medical record (EMR) systems do not provide process-based visualization or real-time interdisciplinary decision support. As a result, clinicians often depend on paper-based tools, fragmented documentation, and manual communication, further hindering timely and coordinated early rehabilitation.

### Study Rationale and Objectives

To address these challenges, we developed the Early Recovery After Traumatic Brain Injury (ERATbi) app, a modular, web-based clinical decision support system (CDSS) designed to assist ICU clinicians in delivering structured early rehabilitation for patients with moderate-to-severe TBI. The system integrates four evidence-based modules: (1) delirium risk management, (2) precision nutrition therapy, (3) stepwise early mobilization, and (4) respiratory care for rib fractures. It incorporates standardized safety thresholds (eg, mean arterial pressure *≥*65 mm Hg, ICP <20 mm Hg), automated alerts, and decision checkpoints to support safe and consistent clinical decision-making.

Grounded in user-centered design and interdisciplinary collaboration, this study describes the ERATbi app’s theoretical framework, system architecture, and simulated clinical workflows, and evaluates its usability, clinical relevance, and potential to enhance safety, standardization, and interdisciplinary coordination in early neurocritical rehabilitation.

## Methods

### System Design and Development Approach

**Figure 1.**
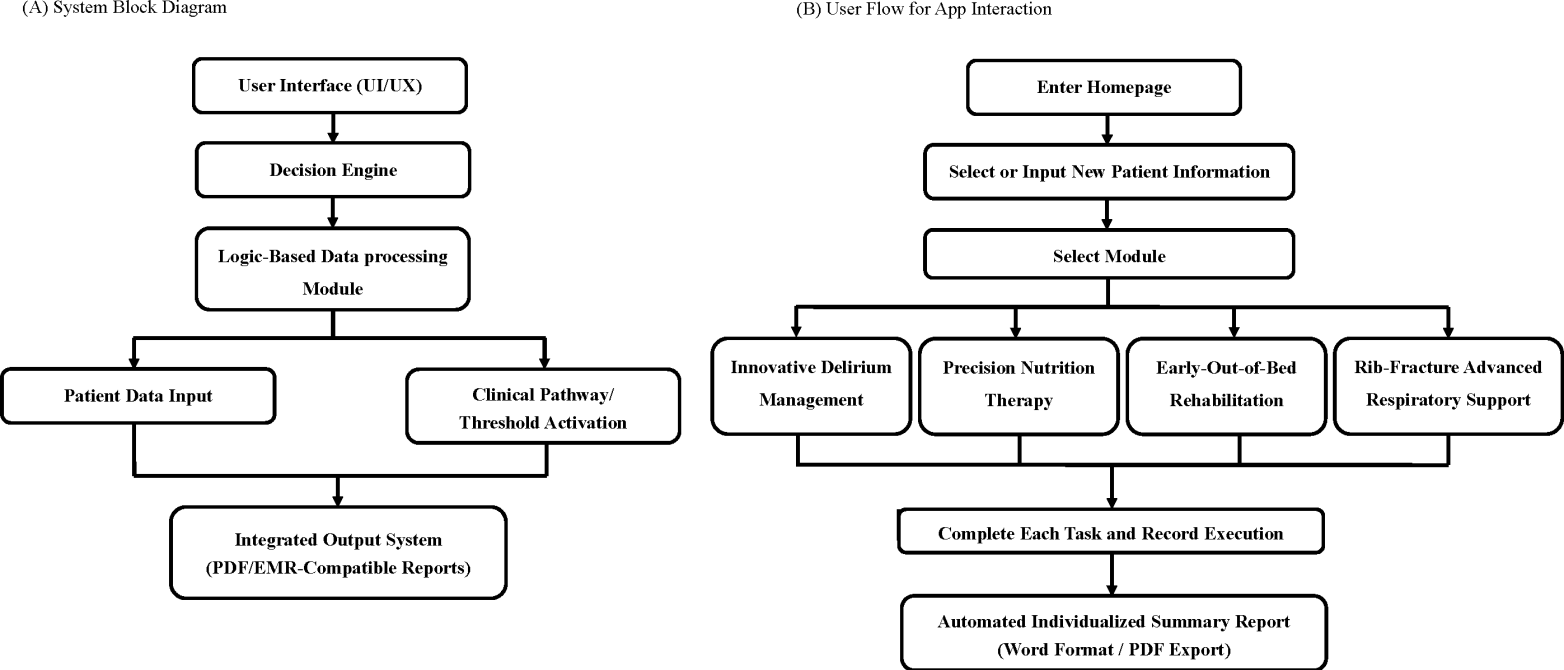
System architecture and user workflow diagram of the ERATbi (Early Recovery After Traumatic Brain Injury) app.

The development of the ERATbi app followed a user-centered design methodology, which is widely recommended for CDSS development in high-acuity settings due to its emphasis on workflow integration, context alignment, and stakeholder engagement [16-18] (Figure 1). A multidisciplinary co-design team—comprising intensivists, physical therapists, dietitians, critical care nurses, and software engineers—collaboratively identified workflow gaps and defined the functional requirements for a neuro-ICU–specific decision-support system. The development process consisted of three iterative stages:

### Needs Assessment Phase

Direct observations and stakeholder interviews were conducted to map existing ICU rehabilitation workflows for patients with moderate-to-severe TBI and to identify barriers to timely intervention. Key gaps included inconsistent interdisciplinary coordination and the absence of real-time patient stability assessment tools.

### Prototype Development Phase

Modular logic trees and interactive wireframes were constructed based on key clinical variables—such as Glasgow Coma Scale (GCS) score, time since admission, vital signs, and device constraints (eg, chest tubes, external ventricular drains). These algorithms were designed to emulate expert reasoning while supporting real-time, safety-focused decision-making.

### Simulated Use Phase

Preliminary usability testing was conducted using mock clinical scenarios, enabling iterative refinement of the interface, navigation structure, and backend decision logic in response to user feedback [17].

### Ethical Considerations

This study did not involve human participants or the collection of personally identifiable information. Usability evaluations were conducted exclusively in a simulated environment using standardized case scenarios developed from deidentified patient data. These data were originally obtained from an institutional review board-approved study at National Taiwan University Hospital (IRB No. 202306107RIND). The original informed consent and IRB approval covered secondary analyses of the existing data without requiring additional consent. All data used in this secondary analysis were deidentified prior to analysis, and no personally identifiable information was accessible to the study team. All participants were ICU clinicians (physicians, nurses, therapists, and dietitians) who evaluated only the app’s interface and workflow. No patient contact occurred, and no clinical interventions were performed. In accordance with institutional policy, the study was deemed exempt from additional IRB review, as it did not constitute human subjects research.

### Modular Architecture and Embedded Clinical Logic

**Figure 2.**
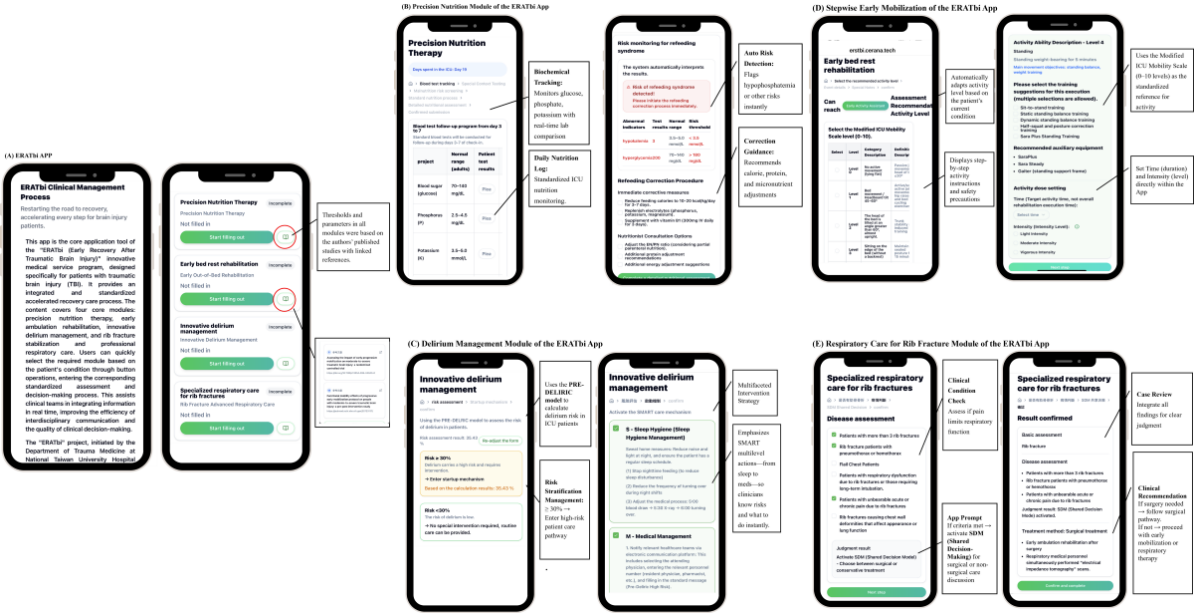
Interface storyboard of the ERATbi (Early Recovery After Traumatic Brain Injury) app demonstrating the stepwise workflow across four functional modules: precision nutrition, delirium management, stepwise early mobilization, and respiratory care.

The ERATbi app consists of four functional modules, each corresponding to a core domain of early neurocritical rehabilitation (Figure 2). This modular structure supports process-based clinical reasoning and enhances interdisciplinary alignment.

### Precision Nutrition Module

This module identifies ICU patients at risk of nutritional deficiency within the first 7 days of admission using real-time GCS and BMI calculations. Consistent with ESPEN (European Society for Clinical Nutrition and Metabolism) and ASPEN (American Society for Parenteral and Enteral Nutrition) guidelines, it stratifies patients into intervention tiers and provides individualized caloric, protein, and micronutrient recommendations [19-21]. Embedded calculators further ensure accurate risk estimation and streamline nutritional decision-making [10,22,23].

### Delirium Management Module

This module incorporates the “Sweet SMART Home” protocol [24] to guide delirium risk stratification based on patient age, cognitive function, and sedation level. It emphasizes nonpharmacologic strategies such as circadian regulation and reorientation, consistent with contemporary ICU guidelines for delirium prevention [11,25].

### Stepwise Early Mobilization Module

Algorithmic logic trees stratify patients according to consciousness level (GCS), physiological thresholds, and device attachments. Based on these criteria, the system generates graded activity recommendations and automated alerts for contraindicated conditions. The framework draws on established ICU mobilization models emphasizing phased recovery and functional readiness [3,26,27].

### Respiratory Care for Rib Fracture Module

This module addresses comorbid thoracic trauma by supporting shared decision-making for surgical fixation and integrating electrical impedance tomography data to guide respiratory management. It includes structured prompts for pulmonary hygiene, analgesia assessment, and individualized weaning plans, aligning with ICU pain and ventilation protocols [28,29].

### User Interface and Decision-Support Design

The ERATbi interface uses a process-based visual workflow that enables clinicians to navigate each module in a structured, stepwise manner. The dashboard displays real-time patient status across the four domains. Core features include auto-calculated clinical thresholds and alert mechanisms triggered by physiological or procedural contraindications.

Although full EMR integration is not yet available, the system generates structured visual summaries and consolidated recommendations to support clinical review. Data-entry fields incorporate real-time validation—such as missing-value alerts and format checks—particularly within the nutrition and mobilization modules. Guided prompts and sequential navigation are designed to reduce cognitive load and enhance data completeness during clinical use.

### Simulation-Based Usability Evaluation

A simulation-based usability evaluation was conducted in the trauma ICU of a tertiary medical center. Eighteen clinicians participated, including physical therapists (n=8), ICU nurses (n=5), attending physicians (n=2), respiratory therapists (n=2), and one dietitian (n=1), all with previous experience in neurocritical care and early mobilization.

Each participant completed two standardized case vignettes reconstructed from de-identified patient records, representing moderate and severe TBI. Each vignette contained five consecutive ICU days of physiological parameters (eg, GCS, MAP [mean arterial pressure], FiO₂ [fraction of inspired oxygen], and ICP), nutritional data, and mobility progression to simulate continuous monitoring. Participants initiated a “new patient entry” and sequentially updated data to reflect daily changes, mirroring real-world decision-making workflows. Although each vignette contained data for multiple ICU days, task-level analysis was based on discrete user interactions, which were counted independently of the number of simulated days.

Case vignettes were tailored to professional scope: physical therapists emphasized activity planning, nurses focused on safety and delirium monitoring, and physicians emphasized interdisciplinary coordination. Participants used hospital-issued tablets or smartphones to complete workflows for at least two modules. All interactions were recorded via screen-capture software for subsequent analysis.

### Assessment Approach

Usability was assessed using a mixed-methods approach integrating quantitative and qualitative data. Quantitative measures included the System Usability Scale (SUS) [30], task completion rate, error rate (eg, incorrect pathway selection, data-entry mistakes), and average task duration. After each simulation, participants completed a customized satisfaction questionnaire with a 5-point Likert scale (Multimedia Appendix 1) and participated in a 10‐15-minute semi-structured interview (Multimedia Appendix 2).

Interviews were conducted face-to-face by a trained observer, audio-recorded with participant consent, and designed to explore usability facilitators, barriers, and improvement opportunities. Example prompts included the following questions. “Which parts of the interface were most intuitive or confusing?” “Were there any steps that slowed your workflow?” “What features would you modify or add to enhance clinical applicability?”

Interview recordings were transcribed verbatim and analyzed thematically following Braun and Clarke’s inductive approach. Two independent researchers coded themes related to facilitators, barriers, and design recommendations. Quantitative and qualitative findings were integrated to develop a comprehensive understanding of user experience and system performance. Sessions were monitored using validated ICU CDSS evaluation frameworks [16,17].

### Methodological Rigor

To further enhance the rigor of this mixed-methods design, several strategies were implemented. For the quantitative component, task-completion metrics and SUS scores were cross-validated by two reviewers to ensure data accuracy. For the qualitative component, two researchers coded the open-ended responses using an inductive thematic approach. Coding discrepancies were discussed and resolved through consensus, and an audit trail was maintained to document analytic decisions.

Data triangulation was achieved by comparing usage metrics, task-performance patterns, and qualitative feedback. Integration of quantitative and qualitative findings followed a convergence model, whereby qualitative themes were compared with performance trends to enhance interpretive depth. Collectively, these approaches strengthened the credibility, dependability, and confirmability of the study.

## Results

### System Architecture Overview

Eighteen ICU professionals participated in the simulation-based usability evaluation using standardized case vignettes. All participants independently completed workflows in at least two ERATbi modules, with their interactions fully recorded for analysis. The ERATbi App integrates four core modules, Delirium Management, Precision Nutrition, Stepwise Early Mobilization, and Respiratory Care, into a unified, logic-driven clinical decision support system tailored for neurocritical rehabilitation. Each module automatically adapts recommendations based on patient-specific variables such as vital signs, level of consciousness (GCS), and device conditions. Safety features include real-time alerting, visual cue signaling deviations from physiological thresholds, and auto-generated clinical summaries. Together, these mechanisms support interdisciplinary coordination and help clinicians maintain a consistent understanding of patient status and safety priorities.

### Usability Metrics

The ERATbi App demonstrated strong usability across all quantitative indicators. The System Usability Scale (SUS) yielded a mean score of 83.6 (SD 7.4), corresponding to the “excellent” usability range [31]. All participants successfully completed their assigned workflows, resulting in a 100% (18/18) task completion rate. The mean time to complete a single module was 6.5 minutes (SD 1.3). The overall error rate was 4.2% (2 errors out of 54 total interactions), primarily involving minor data-entry or selection issues.

User satisfaction, measured via a 5-point Likert scale, averaged 4.7 (SD 0.5), indicating high perceived usefulness and acceptance. Participants consistently highlighted the interface’s logical visual organization, which facilitated intuitive navigation and supported clinical reasoning, particularly beneficial for junior staff. Features such as real-time alerts, automated summaries, and visualized physiological thresholds were frequently cited as improving confidence and enhancing interdisciplinary discussions.

### Qualitative Feedback

Open-ended feedback and observer field notes revealed three major themes: (1) Clarity and cognitive alignment: Participants reported that the stepwise logic structure closely aligned with their real-world ICU decision-making processes, reducing cognitive burden and supporting rapid clinical reasoning; (2) Enhanced interdisciplinary coordination: The integration of rehabilitation, nutrition, delirium, and respiratory considerations within a single interface promoted a shared mental model, enabling more coherent team discussions and planning. In this study, “shared mental model” refers to the alignment of understanding among ICU physicians, nurses, dietitians, and physical therapists regarding safety priorities, patient status, and rehabilitation goals, allowing teams to interpret clinical information consistently and coordinate care more efficiently; (3) Implementation potential: Users expressed confidence in the system’s clinical applicability and highlighted the value of future EMR integration to support handoffs, documentation consistency, and safety review workflows.

Overall, the ERATbi App demonstrated strong feasibility, high user acceptance, and operational efficiency in simulated ICU environments, supporting its potential for broader deployment in neurocritical care workflows.

## Discussion

### Principal Findings

This study developed and evaluated the ERATbi App, a modular CDSS designed to support early rehabilitation planning for ICU patients with moderate-to-severe TBI. In simulation testing with 18 multidisciplinary ICU clinicians, the system demonstrated excellent usability (mean SUS 83.6, SD 7.4), complete task success (100%), a low error rate (4.2%, 2/54 tasks), and high user satisfaction (mean 4.7, SD 0.5). Participants reported that the app’s structured, modular interface enhanced clarity, reduced cognitive burden, and facilitated interdisciplinary communication and real-time decision-making. Collectively, these findings indicate that ERATbi is highly feasible, operationally efficient, and well-positioned for clinical implementation in neurocritical care environments.

### Overcoming the Fragmentation of Early ICU Rehabilitation Support

Prior CDSS research in ICU settings has predominantly focused on single-domain applications, including delirium screening [32,33], primary palliative care [34], rare disease diagnosis [35], fall prevention [36], or early mobility-related decision-making [37,38]. Although these systems have demonstrated benefits within their respective domains, most operate as stand-alone tools lacking interoperability, limiting their ability to support the complex, interdisciplinary workflows required in neurocritical care.

This fragmentation is compounded by EMR-integrated CDSS platforms that largely emphasize documentation or retrospective data retrieval rather than offering adaptive, real-time clinical guidance. These systems rarely adjust recommendations in response to rapidly changing physiological parameters or evolving clinical contexts, reducing their effectiveness in high-acuity decision-making.

To address these persistent gaps, the ERATbi app was conceptualized as a workflow-embedded, multi-domain CDSS specifically tailored for neurocritical care. Its design reflects the multifactorial demands of early rehabilitation in moderate-to-severe TBI—including fluctuating consciousness, invasive device management, physiological instability, and the interactions of multiple disciplines.

Evidence supporting this approach is aligned with recent studies. In 2024, Dunn et al [37] emphasized the need for standardized terminology and safety thresholds in ICU mobility tools, demonstrating high expert agreement (Content Validity Index=0.93). Similarly, in 2025, Wilson-Jene et al [38] demonstrated that algorithm-based guidance improved adherence to safety protocols and reduced mobilization delays. These studies reinforce the necessity of structured, logic-driven, and adaptive CDSS frameworks such as ERATbi.

### Modular Integration and Workflow-Embedded Design in the ERATbi App

The ERATbi app was intentionally engineered as a multidomain CDSS to meet the interdisciplinary and dynamically evolving needs of neurocritical care. Unlike traditional single-domain tools, ERATbi integrates four pillars of early rehabilitation—delirium management, precision nutrition, stepwise mobilization, and respiratory care—within a unified modular interface. This architecture enables clinicians to navigate shifting consciousness levels, invasive device constraints, physiological instability, and interdisciplinary workflows with improved clarity and consistency.

Aligned with evidence-based guidelines and developed using a user-centered design methodology, ERATbi reflects contemporary recommendations that prioritize usability, workflow integration, and team-wide accessibility [39,40]. At the system’s core is a decision engine that synthesizes validated physiological thresholds and individualized risk factors to generate real-time, context-aware recommendations—marking a shift from retrospective documentation tools to proactive, adaptive CDSS design.

Visual logic pathways, real-time alerts, and dynamic thresholds were incorporated to reduce cognitive load while preserving decision accuracy. Prior studies demonstrate that such features improve protocol adherence [41], reduce ICU errors [42], and enhance decision consistency during high-acuity transitions [43]. Additionally, intuitive interfaces with clearly presented thresholds have been shown to strengthen user trust, situational awareness, and adoption across multidisciplinary teams [44-46].

By embedding these technical and human-centered principles within a modular architecture, the ERATbi App provides a scalable and interoperable platform capable of standardizing early rehabilitation workflows and improving safety and communication in complex neurocritical care settings.

### Clinical Implications

This study highlights several important clinical implications. First, the structured, logic-driven design of ERATbi supports consistent clinical decision-making and reduces variability across shifts and providers. Second, its visual dashboards and auto-generated summaries enhance interdisciplinary communication by providing a shared reference for physicians, nurses, dietitians, and physical therapists. Third, the app demonstrates utility as both an educational and an implementation tool, particularly for onboarding new personnel, reinforcing protocol adherence, and supporting quality improvement initiatives. Participants also noted that the modular framework could be adapted to other neurocritical populations, including stroke or post-neurosurgical patients, suggesting the system’s broader clinical scalability.

### Scalability and Data Integration Potential

The ERATbi app was designed for future interoperability with the hospital’s HIS through HL7-FHIR–based data exchange. Structured data entered into the app (eg, GCS, MAP, ICP, FiO₂, mobility level, and nutritional parameters) is planned to be stored using standardized FHIR Observation and Encounter resources. Physiological and biochemical data, such as vital signs, blood gas analyses, and laboratory results, are planned for future automated retrieval from the HIS within the preceding 24‐48 hours to reduce manual input.

Upon full integration, the system is planned to generate AI-assisted summary reports that consolidate the four rehabilitation modules into a single interface. These structured reports are intended to be documented in the EMR and made accessible during interdisciplinary rounds. This planned automated data exchange is expected to improve accuracy, reduce clinician workload, enhance scalability across ICUs, and enable predictive analytics by linking rehabilitation trajectories with clinical outcomes such as mobilization timing, extubation readiness, and ICU length of stay.

### Limitations 

This study has several limitations. First, the evaluation was conducted in a simulation-based environment rather than during real-time clinical deployment, which may affect generalizability. Although case scenarios reflected real moderate-to-severe TBI encounters, certain dynamic or emergent ICU conditions may not have been fully captured. Second, the ERATbi app was assessed as a stand-alone prototype without EMR integration, which may limit immediate scalability and clinical adoption.

### Future Work

Beyond its clinical decision support function, the ERATbi system will be expanded into an educational and quality improvement platform. A planned B2C extension will provide simplified dashboards and interactive modules for patient- and family-centered education to support health literacy and post-ICU self-management. A real-world pilot study is planned at the neuro-ICU of National Taiwan University Hospital, followed by multicenter evaluations examining outcomes such as mobilization timing, delirium incidence, ICU length of stay, and safety events. Future iterations will incorporate a continuous, data-driven feedback mechanism linking clinician decisions with patient outcomes to refine decision thresholds. This iterative framework will enable adaptive modeling and sustained quality improvement in neurocritical rehabilitation.

### Conclusion

The ERATbi app is a modular clinical decision support system designed to address the complex demands of early rehabilitation in neurocritical care. Simulation-based usability testing demonstrated high user acceptance, excellent usability, and strong potential to support workflow standardization, enhance interdisciplinary coordination, and promote patient safety. By integrating evidence-based clinical logic with user-centered design, the system helps to close critical gaps in ICU rehabilitation for patients with moderate-to-severe traumatic brain injury. With planned EMR integration and real-world implementation, the ERATbi app offers a scalable foundation for disseminating early rehabilitation strategies across broader neurocritical care populations.

## Supplementary material

10.2196/79981 Multimedia Appendix 1 ERATbi (Early Recovery After Traumatic Brain Injury) app Usability Evaluation Questionnaire.

10.2196/79981 Multimedia Appendix 2 Qualitative feedback coding framework.

## Abbreviations

CDSS: Clinical Decision Support System
EMR: electronic medical record
ERATbi: Early Recovery After Traumatic Brain Injury
GCS: Glasgow Coma Scale
ICP: intracranial pressure
ICU: intensive care unit
SUS: system usability scale
TBI: traumatic brain injury
