# Supplementary material for: Development of the ERATbi App, a Clinical Decision Support System for Early Recovery After Traumatic Brain Injury in the ICU: Usability Study
Source: JMIR Hum Factors. 2026 Feb 6;13:e79981. doi: 10.2196/79981 (PMC12880592; doi:10.2196/79981)
Supplement: Multimedia Appendix 1 [file humanfactors-v13-e79981-s001.docx]

# Multimedia Appendix 1. ERATbi App Usability Evaluation Questionnaire

Participants were asked to indicate their level of agreement with the following statements based on their experience using the ERATbi App.Scale: 1 = Strongly Disagree, 5 = Strongly Agree.

| No. | Statement | 1 | 2 | 3 | 4 | 5 |
| --- | --- | --- | --- | --- | --- | --- |
| Q1 | The app interface is intuitive and easy to navigate. |  |  |  |  |  |
| Q2 | The stepwise clinical logic reflects real-world ICU decision-making. |  |  |  |  |  |
| Q3 | The integration of rehabilitation, nutrition, and delirium care enhances care coordination. |  |  |  |  |  |
| Q4 | The use of visual cues and real-time alerts supports safe clinical decisions. |  |  |  |  |  |
| Q5 | The system’s modular design is flexible and can be adapted to different patient types. |  |  |  |  |  |
| Q6 | The built-in calculators (e.g., GCS, MUST, BMI) are helpful and easy to use. |  |  |  |  |  |
| Q7 | I would be willing to use this system in real clinical settings. |  |  |  |  |  |
| Q8 | I feel the system saves time during patient assessment and documentation. |  |  |  |  |  |
| Q9 | The app improves interdisciplinary communication in ICU care. |  |  |  |  |  |
| Q10 | Overall, I am satisfied with the usability of the ERATbi App. |  |  |  |  |  |
