# Supplementary material for: Development of the ERATbi App, a Clinical Decision Support System for Early Recovery After Traumatic Brain Injury in the ICU: Usability Study
Source: JMIR Hum Factors. 2026 Feb 6;13:e79981. doi: 10.2196/79981 (PMC12880592; doi:10.2196/79981)
Supplement: Multimedia Appendix 2 [file humanfactors-v13-e79981-s002.docx]

# Multimedia Appendix 2. Qualitative Feedback Coding Framework

The following thematic codes were used for qualitative analysis of open-ended feedback collected after the app simulation.

| Theme Code | Definition | Example Statements |
| --- | --- | --- |
| T1: Interface Clarity | Comments on ease of use, logical layout, and visual comprehension | “I liked how the pathway is structured clearly; it follows my usual thinking.” |
| T2: Workflow Integration | Remarks on how well the app fits into existing clinical routines | “I could imagine using this during ICU rounds—it mirrors our typical flow.” |
| T3: Interdisciplinary Use | Feedback on communication support across disciplines | “It’s helpful that nurses, therapists, and physicians can access the same summary.” |
| T4: Safety and Accuracy | Perceived improvement in safety and protocol compliance | “The alerts prevented me from skipping critical safety steps.” |
| T5: Improvement Suggestions | Specific ideas for feature refinement or integration with hospital systems | “It would be great to link this with our hospital EMR to avoid re-entry of data.” |
